# Modeling Chronic Toxicity: A Comparison of Experimental Variability With (Q)SAR/Read-Across Predictions

**DOI:** 10.3389/fphar.2018.00413

**Published:** 2018-04-25

**Authors:** Christoph Helma, David Vorgrimmler, Denis Gebele, Martin Gütlein, Barbara Engeli, Jürg Zarn, Benoit Schilter, Elena Lo Piparo

**Affiliations:** ^1^In Silico Toxicology Gmbh, Basel, Switzerland; ^2^Data Mining Department, Institute of Computer Science, Johannes Gutenberg Universität Mainz, Mainz, Germany; ^3^Federal Food Safety and Veterinary Office (FSVO), Risk Assessment Division, Bern, Switzerland; ^4^Chemical Food Safety Group, Nestlé Research Center, Lausanne, Switzerland

**Keywords:** (Q)SAR, read-across, LOAEL, experimental variability, lazar

## Abstract

This study compares the accuracy of (Q)SAR/read-across predictions with the experimental variability of chronic lowest-observed-adverse-effect levels (LOAELs) from *in vivo* experiments. We could demonstrate that predictions of the lazy structure-activity relationships (lazar) algorithm within the applicability domain of the training data have the same variability as the experimental training data. Predictions with a lower similarity threshold (i.e., a larger distance from the applicability domain) are also significantly better than random guessing, but the errors to be expected are higher and a manual inspection of prediction results is highly recommended.

## Introduction

Relying on standard animal toxicological testing for chemical hazard identification and characterization is increasingly questioned on both scientific and ethical grounds. In addition, it appears obvious that from a resource perspective, the capacity of standard toxicology to address the safety of thousands of untested chemicals (Fowler et al., 2011) to which human may be exposed is very limited. It has also been recognized that getting rapid insight on toxicity of chemicals in case of emergency safety incidents or for early prioritization in research and development (safety by design) is a big challenge mainly because of the time and cost constraints associated with the generation of relevant animal data. In this context, alternative approaches to obtain timely and fit-for-purpose toxicological information are being developed. Amongst others *in silico* toxicology methods are considered highly promising. Importantly, they are raising more and more interests and getting increased acceptance in various regulatory (e.g., ECHA, 2008; EFSA, 2014, 2016; OECD, 2015; Health Canada, 2016) and industrial (e.g., Lo Piparo et al., 2011; Stanton and Krusezewski, 2016) frameworks.

For a long time already, computational methods have been an integral part of pharmaceutical discovery pipelines, while in chemical food safety their actual potentials emerged only recently (Lo Piparo et al., 2011). In this field, an application considered critical is in the establishment of levels of safety concern in order to rapidly and efficiently manage toxicologically uncharacterized chemicals identified in food. This requires a risk-based approach to benchmark exposure with a quantitative value of toxicity relevant for risk assessment (Schilter et al., 2014). Since chronic studies have the highest power (more animals per group and more endpoints than other studies) and because long-term toxicity studies are often the most sensitive in food toxicology databases, predicting chronic toxicity is of prime importance. Up to now, read-across and Quantitative Structure Activity Relationships (QSAR) have been the most used *in silico* approaches to obtain quantitative predictions of chronic toxicity.

The quality and reproducibility of (Q)SAR and read-across predictions has been a continuous and controversial topic in the toxicological risk-assessment community. Although model predictions can be validated with various procedures, to review results in context of experimental variability has actually been rarely done or attempted. With missing information about the variability of experimental toxicity data it is hard to judge the performance of predictive models objectively and it is tempting for model developers to use aggressive model optimization methods that lead to impressive validation results, but also to overfitted models with little practical relevance.

In the present study, automatic read-across like models were built to generate quantitative predictions of long-term toxicity. The aim of the work was not to predict the nature of the toxicological effects of chemicals, but to obtain quantitative values which could be compared to exposure. Two databases compiling chronic oral rat Lowest Adverse Effect Levels (LOAEL) as endpoint were used. An early review of the databases revealed that many chemicals had at least two independent studies/LOAELs. These studies were exploited to generate information on the reproducibility of chronic animal studies and were used to evaluate prediction performance of the models in the context of experimental variability.

An important limitation often raised for computational toxicology is the lack of transparency on published models and consequently on the difficulty for the scientific community to reproduce and apply them. To overcome these issues, source code for all programs and libraries and the data that have been used to generate this manuscript are made available under GPL3 licenses. Data and compiled programs with all dependencies for the reproduction of results in this manuscript are available as a self-contained docker image. All data, tables, and figures in this manuscript was generated directly from experimental results using the R package knitR.

## Materials and methods

The following sections give a high level overview about algorithms and datasets used for this study. In order to provide unambiguous references to algorithms and datasets, links to source code and data sources are included in the text.

### Datasets

#### Nestlé database

The first database (Nestlé database for further reference) originates from the publication of (Mazzatorta et al., 2008). It contains chronic (>180 days) lowest observed effect levels (LOAEL) for rats (*Rattus norvegicus*) after oral (gavage, diet, drinking water) administration. The Nestlé database consists of 567 LOAEL values for 445 unique chemical structures. The Nestlé database can be obtained from the following GitHub links:

- original data: https://github.com/opentox/loael-paper/blob/revision/data/LOAEL_mg_corrected_smiles_mmol.csv
- unique smiles: https://github.com/opentox/loael-paper/blob/revision/data/mazzatorta.csv
−log10 transfomed LOAEL: https://github.com/opentox/loael-paper/blob/revision/data/mazzatorta_log10.csv.

#### Swiss food safety and veterinary office (FSVO) database

Publicly available data from pesticide evaluations of chronic rat toxicity studies from the European Food Safety Authority (EFSA) (EFSA, 2014), the Joint FAO/WHO Meeting on Pesticide Residues (JMPR) (WHO, 2011), and the US EPA (US EPA, 2011) were compiled to form the FSVO-database. Only studies providing both an experimental NOAEL and an experimental LOAEL were included. The LOAELs were taken as they were reported in the evaluations. Further details on the database are described elsewhere (Zarn et al., 2011, 2013). The FSVO-database consists of 493 rat LOAEL values for 381 unique chemical structures. It can be obtained from the following GitHub links:

- original data: https://github.com/opentox/loael-paper/blob/revision/data/NOAEL-LOAEL_SMILES_rat_chron.csv
- unique smiles and mmol/kg_bw/day units: https://github.com/opentox/loael-paper/blob/revision/data/swiss.csv
−log10 transfomed LOAEL: https://github.com/opentox/loael-paper/blob/revision/data/swiss_log10.csv

#### Preprocessing

Chemical structures (represented as SMILES; Weininger, 1988) in both databases were checked for correctness. When syntactically incorrect or missing SMILES were generated from other identifiers (e.g., names, CAS numbers). Unique smiles from the OpenBabel library (O'Boyle et al., 2011) were used for the identification of duplicated structures.

Studies with undefined or empty LOAEL entries were removed from the databases. LOAEL values were converted to mmol/kg bw/day units and rounded to five significant digits. For prediction, validation, and visualization purposes −log10 transformations are used.

#### Derived datasets

Two derived datasets were obtained from the original databases:

The *test* dataset contains data from compounds that occur in both databases. LOAEL values equal at five significant digits were considered as duplicates originating from the same study/publication and only one instance was kept in the test dataset. The test dataset has 375 LOAEL values for 155 unique chemical structures and was used for

- evaluating experimental variability
- comparing model predictions with experimental variability.

The *training* dataset is the union of the Nestlé and the FSVO databases and it was used to build predictive models. LOAEL duplicates were removed using the same criteria as for the test dataset. The training dataset has 998 LOAEL values for 671 unique chemical structures.

### Algorithms

In this study we are using the modular lazar (*la*zy *s*tructure *a*ctivity *r*elationships) framework (Maunz et al., 2013) for model development and validation. The complete lazar source code can be found on GitHub.

lazar follows the following basic workflow:

For a given chemical structure lazar

- searches in a database for similar structures (*neighbors*) with experimental data,
- builds a local QSAR model with these neighbors and
- uses this model to predict the unknown activity of the query compound.

This procedure resembles an automated version of *read across* predictions in toxicology, in machine learning terms it would be classified as a *k-nearest-neighbor* algorithm.

Apart from this basic workflow lazar is completely modular and allows the researcher to use any algorithm for similarity searches and local QSAR modeling. Algorithms used within this study are described in the following sections.

#### Neighbor identification

Similarity calculations are based on MolPrint2D fingerprints (Bender et al., 2004) from the OpenBabel chemoinformatics library (O'Boyle et al., 2011).

The MolPrint2D fingerprint uses atom environments as molecular representation, which resemble basically the chemical concept of functional groups. For each atom in a molecule it represents the chemical environment using the atom types of connected atoms.

MolPrint2D fingerprints are generated dynamically from chemical structures and do not rely on predefined lists of fragments (such as OpenBabel FP3, FP4, or MACCs fingerprints or lists of toxocophores/toxicophobes). This has the advantage that they may capture substructures of toxicological relevance that are not included in other fingerprints.

From MolPrint2D fingerprints we can construct a feature vector with all atom environments of a compound, which can be used to calculate chemical similarities.

The chemical similarity between two compounds A and B is expressed as the proportion between atom environments common in both structures *A* ∩ *B* and the total number of atom environments *A* ∪ *B* (Jaccard/Tanimoto index, Equation 1).

(1) $$\begin{array}{l} sim=\frac{\left|A\cap B\right|}{\left|A\cup B\right|} \end{array}$$

The threshold selection is a trade-off between prediction accuracy (high threshold) and the number of predictable compounds (low threshold). As it is in many practical cases desirable to make predictions even in the absence of closely related neighbors, we follow a tiered approach:

- First a similarity threshold of 0.5 is used to collect neighbors, to create a local QSAR model and to make a prediction for the query compound.
- If any of these steps fails, the procedure is repeated with a similarity threshold of 0.2 and the prediction is flagged with a warning that it might be out of the applicability domain of the training data.
- Similarity thresholds of 0.5 and 0.2 are the default values chosen by the software developers and remained unchanged during the course of these experiments.

Compounds with the same structure as the query structure are automatically eliminated from neighbors to obtain unbiased predictions in the presence of duplicates.

#### Local QSAR models and predictions

Only similar compounds (*neighbors*) above the threshold are used for local QSAR models. In this investigation we are using weighted random forests regression (RF) for the prediction of quantitative properties. First all uninformative fingerprints (i.e., features with identical values across all neighbors) are removed. The remaining set of features is used as descriptors for creating a local weighted RF model with atom environments as descriptors and model similarities as weights. The RF method from the caret R package (Kuhn, 2008) is used for this purpose. Models are trained with the default caret settings, optimizing the number of RF components by bootstrap resampling.

Finally the local RF model is applied to predict the activity of the query compound. The root-mean-square error (RMSE) of bootstrapped local model predictions is used to construct 95% prediction intervals at 1.96^*^RMSE. The width of the prediction interval indicates the expected prediction accuracy. The “true” value of a prediction should be with 95% probability within the prediction interval.

If RF modeling or prediction fails, the program resorts to using the weighted mean of the neighbors LOAEL values, where the contribution of each neighbor is weighted by its similarity to the query compound. In this case the prediction is also flagged with a warning.

#### Applicability domain

The applicability domain (AD) of lazar models is determined by the structural diversity of the training data. If no similar compounds are found in the training data no predictions will be generated. Warnings are issued if the similarity threshold has to be lowered from 0.5 to 0.2 in order to enable predictions and if lazar has to resort to weighted average predictions, because local random forests fail. Thus, predictions without warnings can be considered as close to the applicability domain and predictions with warnings as more distant from the applicability domain. Quantitative applicability domain information can be obtained from the similarities of individual neighbors.

Local regression models consider neighbor similarities to the query compound, by weighting the contribution of each neighbor is by similarity. The variability of local model predictions is reflected in the 95% prediction interval associated with each prediction.

#### Validation

For the comparison of experimental variability with predictive accuracies we are using a test set of compounds that occur in both databases. Unbiased read across predictions are obtained from the *training* dataset, by removing *all* information from the test compound from the training set prior to predictions. This procedure is hardcoded into the prediction algorithm in order to prevent validation errors. As we have only a single test set no model or parameter optimizations were performed in order to avoid overfitting a single dataset.

Results from 50 repeated 10-fold crossvalidations with independent training/test set splits are provided as additional information to the test set results.

The final model for production purposes was trained with all available LOAEL data (Nestlé and FSVO databases combined).

### Availability

**Figure 1:**
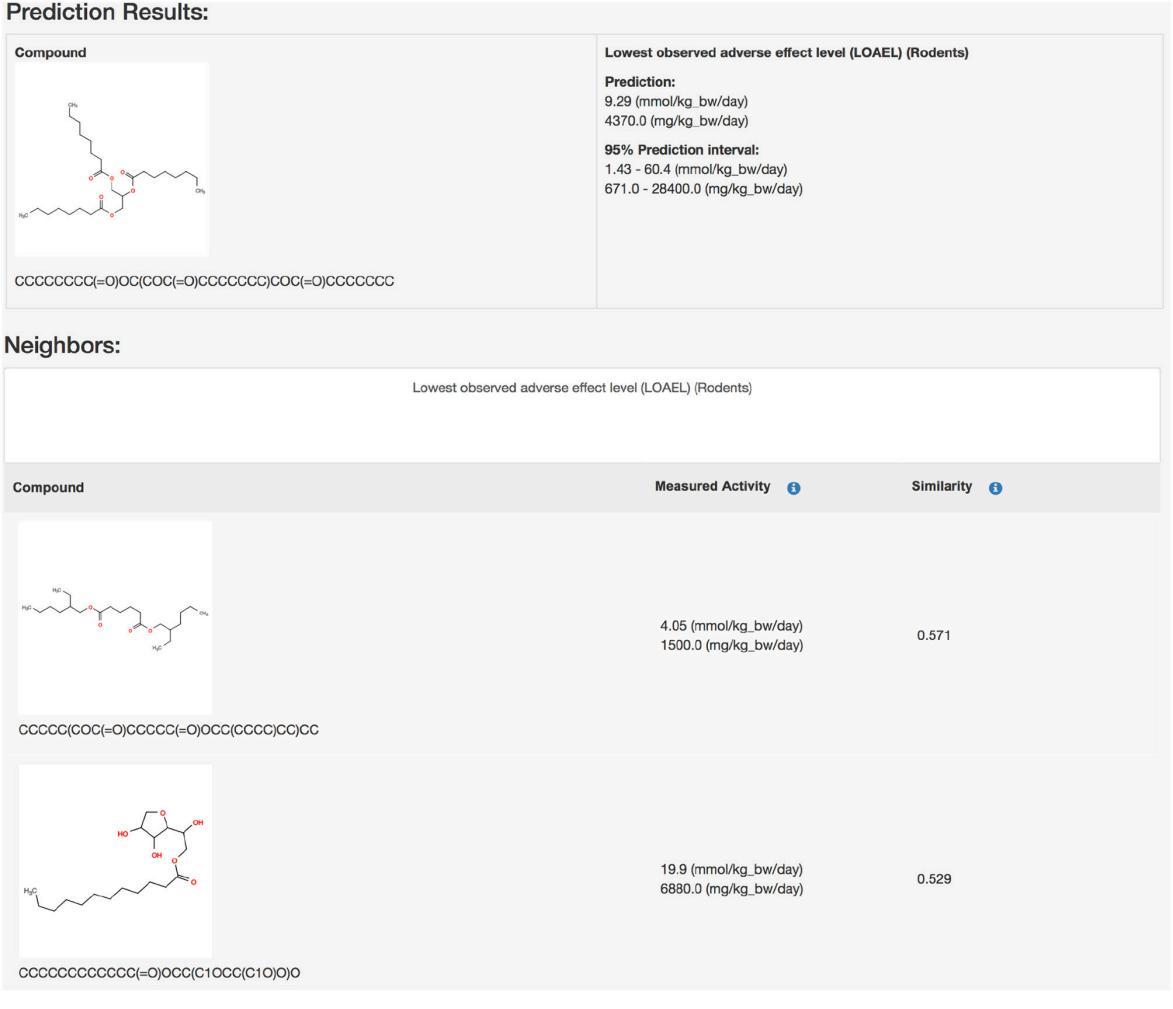
Screenshot of a lazar prediction from the public webinterface.

**Public webinterface** https://lazar.in-silico.ch (see Figure 1)
**lazar framework** https://github.com/opentox/lazar (source code)
**lazar GUI** https://github.com/opentox/lazar-gui (source code)
**Manuscript** https://github.com/opentox/loael-paper/tree/revision (source code for the manuscript and validation experiments)
**Docker image** https://hub.docker.com/r/insilicotox/loael-paper/ (container with manuscript, validation experiments, lazar libraries, and third party dependencies)

## Results

### Dataset comparison

The main objective of this section is to compare the content of both databases in terms of structural composition and LOAEL values, to estimate the experimental variability of LOAEL values and to establish a baseline for evaluating prediction performance.

### Structural diversity

**Figure 2:**
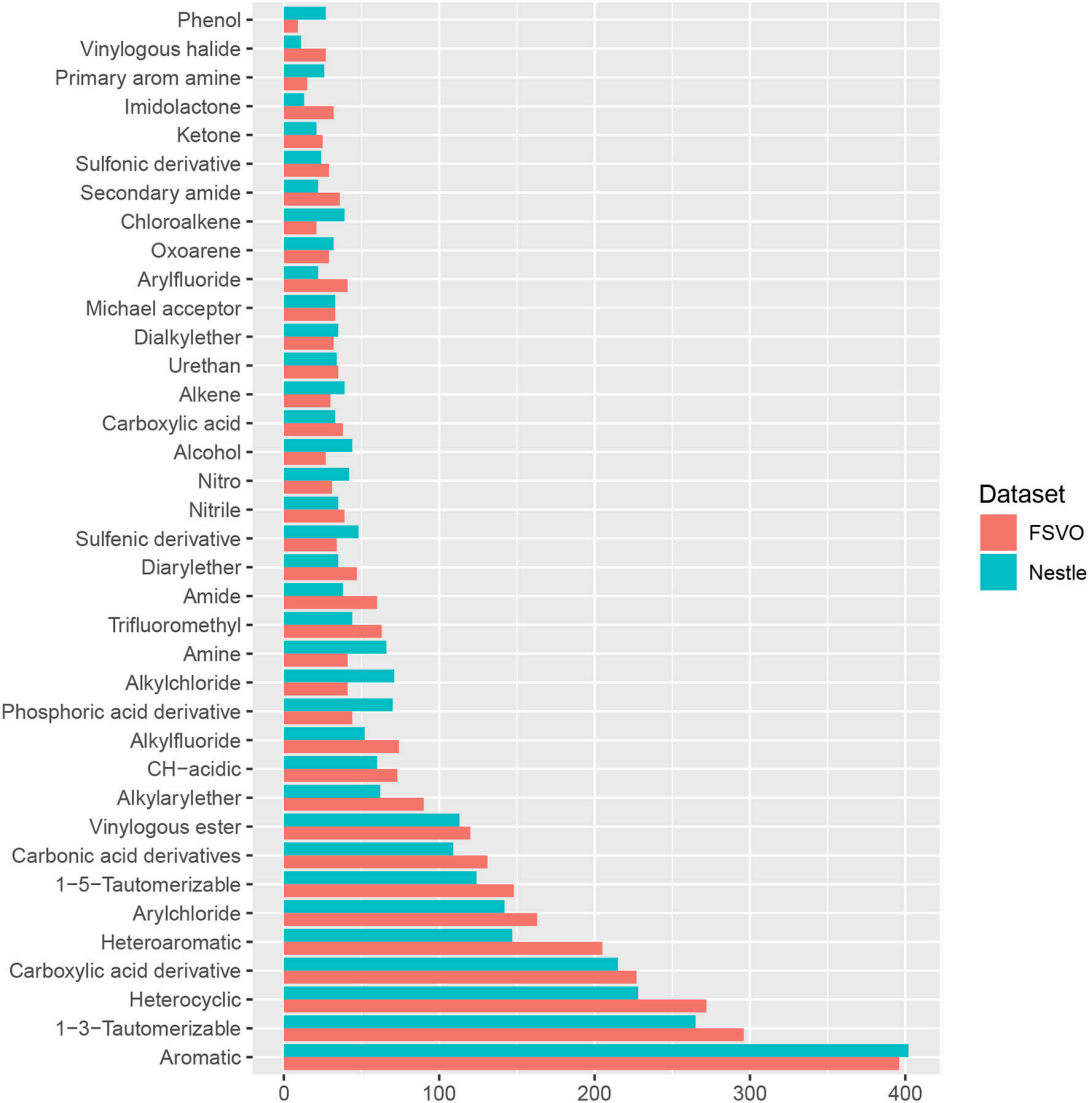
Frequency of functional groups.

In order to compare the structural diversity of both databases we evaluated the frequency of functional groups from the OpenBabel FP4 fingerprint. Figure 2 shows the frequency of functional groups in both databases. One hundred and thirty-nine functional groups with a frequency > 25 are depicted, the complete table for all functional groups can be found in the supplemental material at GitHub.

This result was confirmed with a visual inspection using the CheS-Mapper (Chemical Space Mapping and Visualization in 3D, Gütlein et al., 2012) tool. CheS-Mapper can be used to analyze the relationship between the structure of chemical compounds, their physico-chemical properties, and biological or toxic effects. It depicts closely related (similar) compounds in 3D space and can be used with different kinds of features. We have investigated structural as well as physico-chemical properties and concluded that both databases are very similar, both in terms of chemical structures and physico-chemical properties.

The only statistically significant difference between both databases is that the Nestlè database contains more small compounds (61 structures with less than 11 non-hydrogen atoms) than the FSVO-database (19 small structures, chi-square test: *p*-value 3.7E-7).

### Experimental variability vs. prediction uncertainty

Duplicated LOAEL values can be found in both databases and there is a substantial number of 155 compounds with more than one LOAEL. These chemicals allow us to estimate the variability of experimental results within individual databases and between databases. Data with *identical* values (at five significant digits) in both databases were excluded from variability analysis, because it it likely that they originate from the same experiments.

#### Intra database variability

Both databases contain substances with multiple measurements, which allow the determination of experimental variabilities. For this purpose we have calculated the mean LOAEL standard deviation of compounds with multiple measurements. Mean standard deviations and thus experimental variabilities are similar for both databases.

The Nestlé database has 567 LOAEL values for 445 unique structures, 93 compounds have multiple measurements with a mean standard deviation (−log10 transformed values) of 0.32 (0.56 mg/kg_bw/day, 0.56 mmol/kg_bw/day) (Mazzatorta et al., 2008, Figure 3).

**Figure 3:**
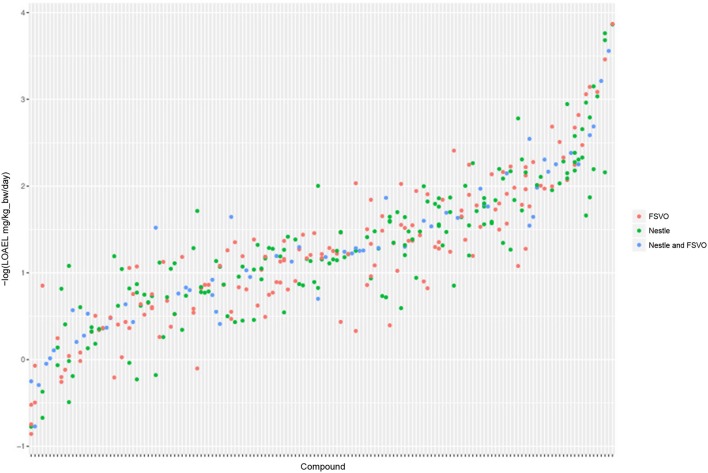
LOAEL distribution and variability of compounds with multiple measurements in both databases. Compounds were sorted according to LOAEL values. Each vertical line represents a compound, and each dot an individual LOAEL value. Experimental variability can be inferred from dots (LOAELs) on the same line (compound).

The FSVO database has 493 rat LOAEL values for 381 unique structures, 91 compounds have multiple measurements with a mean standard deviation (−log10 transformed values) of 0.29 (0.57 mg/kg_bw/day, 0.59 mmol/kg_bw/day) (Figure 3).

Standard deviations of both databases do not show a statistically significant difference with a *p*-value (*t*-test) of 0.21. The combined test set has a mean standard deviation (−log10 transformed values) of 0.33 (0.56 mg/kg_bw/day, 0.55 mmol/kg_bw/day) (Figure 3).

#### Inter database variability

In order to compare the correlation of LOAEL values in both databases and to establish a reference for predicted values, we have investigated compounds, that occur in both databases.

**Figure 4:**
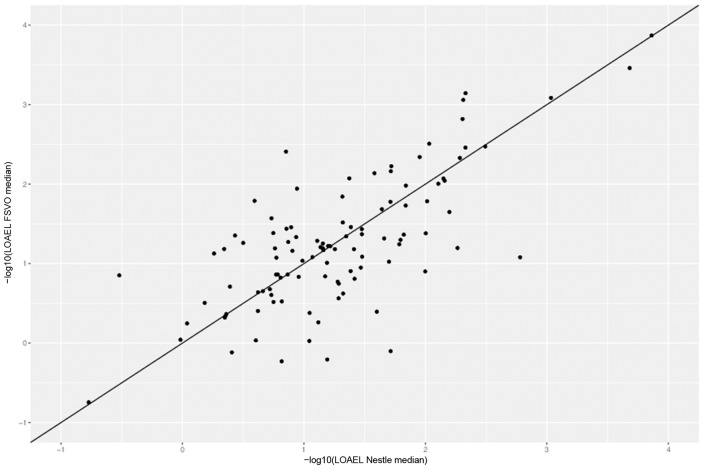
Correlation of median LOAEL values from Nestlé and FSVO databases. Data with identical values in both databases was removed from analysis.

Figure 4 depicts the correlation between LOAEL values from both databases. As both databases contain duplicates medians were used for the correlation plot and statistics. It should be kept in mind that the aggregation of duplicated measurements into a single median value hides a substantial portion of the experimental variability. Correlation analysis shows a significant (*p*-value < 2.2e-16) correlation between the experimental data in both databases with r^2: 0.52, RMSE: 0.59.

**Figure 5:**
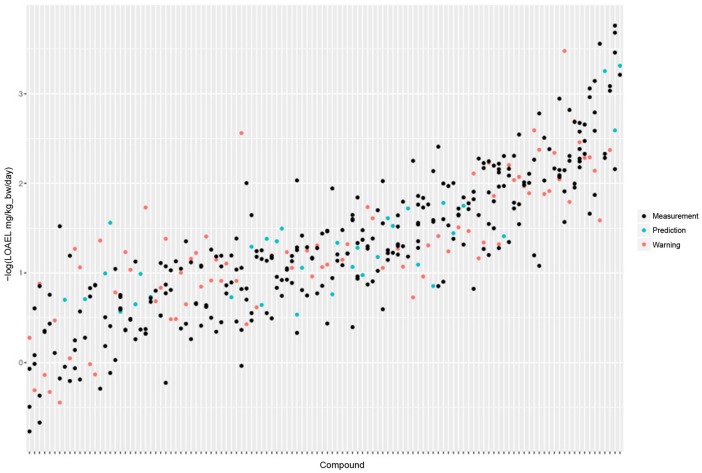
Comparison of experimental with predicted LOAEL values. Each vertical line represents a compound, dots are individual measurements (blue), predictions (green), or predictions far from the applicability domain, i.e., with warnings (red).

Figure 5 shows the experimental LOAEL variability of compounds occurring in both datasets (i.e., the *test* dataset) colored in blue (experimental). This is the baseline reference for the comparison with predicted values.

### Local QSAR models

In order to compare the performance of *in silico* read across models with experimental variability we used compounds with multiple measurements as a test set (375 measurements, 155 compounds). lazar read across predictions were obtained for 155 compounds, 37 predictions failed, because no similar compounds were found in the training data (i.e., they were not covered by the applicability domain of the training data).

In 100% of the test examples experimental LOAEL values were located within the 95% prediction intervals.

Figure 5 shows a comparison of predicted with experimental values. Most predicted values were located within the experimental variability.

**Figure 6:**
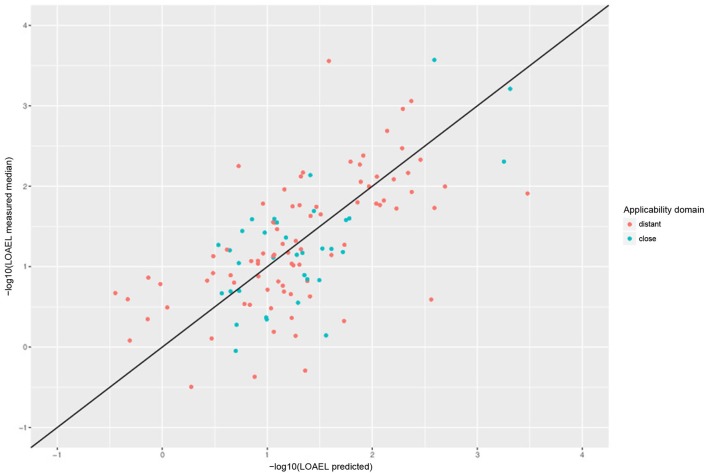
Correlation of experimental with predicted LOAEL values (test set). Green dots indicate predictions close to the applicability domain (i.e., without warnings), red dots indicate predictions far from the applicability domain (i.e., with warnings).

Correlation analysis was performed between individual predictions and the median of experimental data. All correlations are statistically highly significant with a *p*-value < 2.2e-16. These results are presented in Figure 6 and Table 1. Please bear in mind that the aggregation of multiple measurements into a single median value hides experimental variability.

**Table 1:** Comparison of model predictions with experimental variability.

**Comparison**	***r*^2^**	**RMSE**	**Nr. predicted**
Nestlé vs. FSVO database	0.52	0.59
AD close predictions vs. test median	0.48	0.56	34/155
AD distant predictions vs. test median	0.38	0.68	84/155
All predictions vs. test median	0.4	0.65	118/155

**Figure 7:**
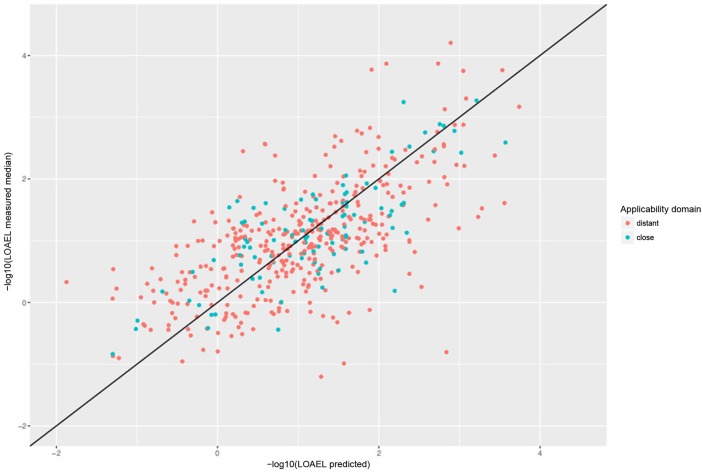
Correlation of predicted vs. measured values from a randomly selected crossvalidation with MP2D fingerprint descriptors and local random forest models.

For a further assessment of model performance three independent 10-fold cross-validations were performed. Results are summarized in Table 2 and Figure 7. All correlations of predicted with experimental values are statistically highly significant with a *p*-value < 2.2e-16. This was observed for compounds close and more distant to the applicability domain.

**Table 2:** Results (mean and standard deviation) from 50 independent 10-fold crossvalidations.

**Predictions**	***r*^2^**	**RMSE**	**Nr. predicted**
AD close	0.6 ± 0.04	0.58 ± 0.02	97 ± 4
AD distant	0.43 ± 0.01	0.8 ± 0.01	380 ± 5
All	0.46 ± 0.01	0.76 ± 0.01	477 ± 4

## Discussion

It is currently acknowledged that there is a strong need for toxicological information on the multiple thousands of chemicals to which human may be exposed through food. These include for example many chemicals in commerce, which could potentially find their way into food (Fowler et al., 2011; Stanton and Krusezewski, 2016), but also substances migrating from food contact materials (Grob et al., 2006), chemicals generated over food processing (Cotterill et al., 2008), environmental contaminants as well as inherent plant toxicants (Schilter et al., 2013). For the vast majority of these chemicals, no toxicological data is available and consequently insight on their potential health risks is very difficult to obtain. It is recognized that testing all of them in standard animal studies is neither feasible from a resource perspective nor desirable because of ethical issues associated with animal experimentation. In addition, for many of these chemicals, risk may be very low and therefore testing may actually be irrelevant. In this context, the identification of chemicals of most concern on which limited resource available should focused is essential and computational toxicology is thought to play an important role for that.

In order to establish the level of safety concern of food chemicals toxicologically not characterized, a methodology mimicking the process of chemical risk assessment, and supported by computational toxicology, was proposed (Schilter et al., 2014). It is based on the calculation of margins of exposure (MoE) that is the ratio between the predicted chronic toxicity value (LOAEL) and exposure estimate. The level of safety concern of a chemical is then determined by the size of the MoE and its suitability to cover the uncertainties of the assessment. To be applicable, such an approach requires quantitative predictions of toxicological endpoints relevant for risk assessment. The present work focuses on the prediction of chronic toxicity, a major and often pivotal endpoint of toxicological databases used for hazard identification and characterization of food chemicals.

In a previous study, automated read-across like models for predicting carcinogenic potency were developed. In these models, substances in the training dataset similar to the query compounds are automatically identified and used to derive a quantitative TD50 value. The errors observed in these models were within the published estimation of experimental variability (Lo Piparo et al., 2014). In the present study, a similar approach was applied to build models generating quantitative predictions of long-term toxicity. Two databases compiling chronic oral rat lowest adverse effect levels (LOAEL) as reference value were available from different sources. Our investigations clearly indicated that the Nestlé and FSVO databases are very similar in terms of chemical structures and properties as well as distribution of experimental LOAEL values. The only significant difference that we observed was that the Nestlé one has larger amount of small molecules, than the FSVO database. For this reason we pooled both databases into a single training dataset for read across predictions.

An early review of the databases revealed that 155 out of the 671 chemicals available in the training datasets had at least two independent studies/LOAELs. These studies were exploited to generate information on the reproducibility of chronic animal studies and were used to evaluate prediction performance of the models in the context of experimental variability. Considerable variability in the experimental data was observed. Study design differences, including dose selection, dose spacing, and route of administration are likely explanation of experimental variability. High experimental variability has an impact on model building and on model validation. First it influences model quality by introducing noise into the training data, secondly it influences accuracy estimates because predictions have to be compared against noisy data where “true” experimental values are unknown. This will become obvious in the next section, where comparison of predictions with experimental data is discussed. The data obtained in the present study indicate that lazar generates reliable predictions for compounds within the applicability domain of the training data (i.e., predictions without warnings, which indicates a sufficient number of neighbors with similarity > 0.5 to create local random forest models). Correlation analysis shows that errors (RMSE) and explained variance (*r*^2^) are comparable to experimental variability of the training data.

Predictions with a warning (neighbor similarity < 0.5 and > 0.2 or weighted average predictions) are more uncertain. However, they still show a strong correlation with experimental data, but the errors are ~ 20–40% larger than for compounds within the applicability domain (Figure 6 and Table 2). Expected errors are displayed as 95% prediction intervals, which covers 100% of the experimental data. The main advantage of lowering the similarity threshold is that it allows to predict a much larger number of substances than with more rigorous applicability domain criteria. As each of this prediction could be problematic, they are flagged with a warning to alert risk assessors that further inspection is required. This can be done in the graphical interface (https://lazar.in-silico.ch) which provides intuitive means of inspecting the rationales and data used for read across predictions.

Finally there is a substantial number of chemicals (37), where no predictions can be made, because no similar compounds in the training data are available. These compounds clearly fall beyond the applicability domain of the training dataset and in such cases predictions should not be used. In order to expand the domain of applicability, the possibility to design models based on shorter, less than chonic studies should be studied. It is likely that more substances reflecting a wider chemical domain may be available. To predict such shorter duration endpoints would also be valuable for chronic toxicy since evidence suggest that exposure duration has little impact on the levels of NOAELs/LOAELs (Zarn et al., 2011, 2013).

### Lazar predictions

Tables 1, 2 and Figures 5–7 clearly indicate that lazar generates reliable predictions for compounds within the applicability domain of the training data (i.e., predictions without warnings, which indicates a sufficient number of neighbors with similarity > 0.5 to create local random forest models). Correlation analysis (Tables 1, 2) shows, that errors (*RMSE*) and explained variance (*r*^2^) are comparable to experimental variability of the training data.

Predictions with a warning (neighbor similarity < 0.5 and > 0.2 or weighted average predictions) are a gray zone. They still show a strong correlation with experimental data, but the errors are larger than for compounds within the applicability domain (Tables 1, 2). Expected errors are displayed as 95% prediction intervals, which covers 100% of the experimental data. The main advantage of lowering the similarity threshold is that it allows to predict a much larger number of substances than with more rigorous applicability domain criteria. As each of this prediction could be problematic, they are flagged with a warning to alert risk assessors that further inspection is required. This can be done in the graphical interface (https://lazar.in-silico.ch) which provides intuitive means of inspecting the rationales and data used for read across predictions.

## Summary

In conclusion, we could demonstrate that lazar predictions within the applicability domain of the training data have the same variability as the experimental training data. In such cases experimental investigations can be substituted with *in silico* predictions. Predictions with a lower similarity threshold can still give usable results, but the errors to be expected are higher and a manual inspection of prediction results is highly recommended. Anyway, our suggested workflow includes always the visual inspection of the chemical structures of the neighbors selected by the model. Indeed it will strength the prediction confidence (if the input structure looks very similar to the neighbors selected to build the model) or it can drive to the conclusion to use read-across with the most similar compound of the database (in case not enough similar compounds to build the model are present in the database).

## Author contributions

CH: Study design and coordination, scientific programming, computational experiments. DV and DG: Scientific programming, computational experiments, GUI design and implementation. MG: Database comparison. BE and JZ: FSVO database. BS: Nestle database, scientific supervision. EL: Study design and coordination, Nestle database.

### Conflict of interest statement

BS and EL were employed by company Nestlé Research Center. CH, DV, and DG were employed by company in silico toxicology gmbh. This study was funded in part by Nestlé. FSVO shared their data and expertise without any financial support. The other authors declare that the research was conducted in the absence of any commercial or financial relationships that could be construed as a potential conflict of interest.

## References

[B1] Bender A. Mussa H. Y. Glen R. C. Reiling S. (2004). Molecular similarity searching using atom environments, information-based feature selection, and a Naïve Bayesian Classifier. J. Chem. Inform. Comput. Sci. 44, 170–178. 10.1021/ci034207y 14741025

[B2] Cotterill J. V. Chaudry M. Q. Mattews W. Watkins R. W. (2008). *In silico* assessment of toxicity of heat-generated food contaminants. Food Chem. Toxicol. 46, 1905–1918. 10.1016/j.fct.2008.01.030 18334278

[B3] ECHA (2008). Guidance on Information Requirements and Chemical Safety Assessment, Chapter R.6: QSARs and Grouping of Chemicals. ECHA.

[B4] EFSA (2014). Rapporteur Member State Assessment Reports Submitted for the EU Peer Review of Active Substances Used in Plant Protection Products. Available online at: http://dar.efsa.europa.eu/dar-web/provision

[B5] EFSA (2016). Guidance on the establishment of the residue definition for dietary assessment: EFSA panel on Plant Protect Products and Their Residues (PPR). EFSA J. 14, 1–12. 10.2903/j.efsa.2016.4549

[B6] Fowler B. Savage S. Mendez B. (2011). White Paper: Protecting Public Health in the 21st Century: The Case for Computational Toxicology. ICF International Inc. Available online at: https://www.icf.com/.

[B7] Grob K. Biedermann M. Scherbaum E. Roth M. Rieger K. (2006). Food contamination with organic materials in perspective: packaging materials as the largest and least controlled source? A view focusing on the European situation. Crit. Rev. Food. Sci. Nutr. 46, 529–535. 10.1080/10408390500295490 16954061

[B8] Gütlein M. Karwath A. Kramer S. (2012). CheS-Mapper - chemical space mapping and visualization in 3D. J. Cheminformatics 4:7. 10.1186/1758-2946-4-7 22424447 PMC3331825

[B9] Health Canada (2016). Available online at: https://www.canada.ca/en/health-canada/services/chemical-substances/chemicals-management-plan.html

[B10] Kuhn M. (2008). Building predictive models in R using the caret package. J. Stat. Soft. 28, 1–26. 10.18637/jss.v028.i05

[B11] Lo Piparo E. Maunz A. Helma C. Vorgrimmler D. Schilter B. (2014). Automated and reproducible read-across like models for predicting carcinogenic potency. Regul. Toxicol. Pharmacol. 70, 370–378. 10.1016/j.yrtph.2014.07.010 25047023

[B12] Lo Piparo E. Worth A. Manibusan A. Yang C. Schilter B. Mazzatorta P.. (2011). Use of computational tools in the field of food safety. Regul. Toxicol. Pharmacol. 60, 354–362. 10.1016/j.yrtph.2011.05.003 21600952

[B13] Maunz A. Gütlein M. Rautenberg M. Vorgrimmler D. Gebele D. Helma C. (2013). Lazar: a modular predictive toxicology framework. Front. Pharmacol. 4:38. 10.3389/fphar.2013.00038 23761761 PMC3669891

[B14] Mazzatorta P. Estevez M. D. Coulet M. B. Schilter B. (2008). Modeling oral rat chronic toxicity. J. Chem. Inform. Model. 48, 1949–1954. 10.1021/ci8001974 18803370

[B15] O'Boyle N. M. Banck M. James C. A. Morley C. Vandermeersch T. Hutchison G. R. (2011). Open Babel: an open chemical toolbox. J. Cheminformatics 3:33. 10.1186/1758-2946-3-33 21982300 PMC3198950

[B16] OECD (2015). Fundamental and guiding principles for (Q)SAR analysis of chemicals carcinogens with mechanistic considerations monograph 229 ENV/JM/MONO(2015)46, in Series on Testing and Assessment No 229 (Paris).

[B17] Schilter B. Benigni R. Boobis A. Chiodini A. Cockburn A. Cronin M. T.. (2014). Establishing the level of safety concern for chemicals in food without the need for toxicity testing. Regul. Toxicol. Pharmacol. 68, 275–298. 10.1016/j.yrtph.2013.08.018 24012706

[B18] Schilter B. Constable A. Perrin I. (2013). Naturally occurring toxicants of plant origin: risk assessment and management considerations. in Food Safety Management: A Practical Guide for Industry, ed Motarjemi Y. (Amsterdam: Elsevier), 45–57.

[B19] Stanton K. Krusezewski F. H. (2016). Quantifying the benefits of using read-across and *in silico* techniques to fullfill hazard data requirements for chemical categories. Regul. Toxicol. Pharmacol. 81, 250–259. 10.1016/j-yrtph.2016.09.004 27612993

[B20] US EPA (2011). Fact Sheets on New Active Ingredients.

[B21] Weininger D. (1988). SMILES, a chemical language and information system. 1. Introduction to methodology and encoding rules. J. Chem. Inform. Comput. Sci. 28, 31–36. 10.1021/ci00057a005

[B22] WHO (2011). Joint FAO/WHO Meeting on Pesticide Residues (JMPR)Publications. Available online at: http://www.who.int/foodsafety/publications/jmpr-monographs/en/

[B23] Zarn J. A. Engeli B. E. Schlatter J. R. (2011). Study parameters influencing NOAEL and LOAEL in toxicity feeding studies for pesticides:exposure duration versus dose decrement, dose spacing, group size and chemical class. Regul. Toxicol. Pharmacol. 61, 243–250. 10.1016/j.yrtph.2011.08.004 21875639

[B24] Zarn J. A. Engeli B. E. Schlatter J. R. (2013). Characterization of the dose decrement in regulatory rat pesticide toxicity feeding studies. Regul. Toxicol. Pharmacol. 67, 215–220. 10.1016/j.yrtph.2013.07.012 23911766

